# Tracheostomy in Spinal Cord Injured Patients

**Published:** 2011-10-17

**Authors:** Javier-Romero Ganuza, Antonio Oliviero

**Affiliations:** Center: ICU and Neurology Unit, Internal Medicine Department, National Hospital for Paraplegics, Toledo, Spain.

**Keywords:** Spinal Cord Injury, Tracheostomy, Respiratory Failure, Mechanical Ventilation

## Abstract

Patients with cervical spinal cord injury frequently need prolonged mechanical ventilation as a result of worsening pulmonary vital capacity due to paralysis of respiratory muscles, severe impairment of tracheobronchial secretions clearance and high incidence of respiratory complications like pneumonia or atelectasis. Patients with thoracic spinal cord injury may need mechanical ventilation due to associate injuries. For these reasons, tracheostomy is frequently performed in these patients, more frequently when the spinal cord injury is at cervical level.

Percutaneous technique, performed in the ICU, should be considered the preferred procedure for performing elective tracheostomies in spinal cord injured patients. Tracheostomy should be implemented as soon as possible in SCI patients they require prolonged mechanical ventilation. Tracheostomy can be performed just after anterolateral cervical spine fixation surgery. Tracheostomy can be removed when no longer needed without major complications.

## INTRODUCTION

It is estimated that approximately 40 new traumatic spinal cord injuries are produced annually per million population in the U. S. Approximately half of these patients suffer cervical spinal cord injury (SCI) 1. Respiratory insufficiency is the most common early complication and the primary cause of death both in the acute and chronic phases of recovery from SCI 2–4. An injury in cervical spinal cord causes tetraplegia and prolonged respiratory failure due to paralysis of the respiratory muscles, through to loss of function of the corticospinal ways and the second motor neuron injury 5.

Patients with cervical SCI frequently need prolonged mechanical ventilation as a result of worsening pulmonary vital capacity due to paralysis of respiratory muscles, severe impairment of tracheobronchial secretions clearance and high incidence of respiratory complications like pneumonia or atelectasis, 3, 6–9. For these reasons, tracheostomy is frequently performed in these patients 5,10. Tracheostomy is performed more frequently when the spinal cord injury is at cervical level. It is directly correlated with the level of the injury and the degree of motor completeness in cervical spinal cord injured patients, and related with direct chest trauma in thoracic spinal cord injured patients 3,4.

## THE TRACHEOSTOMY

Tracheostomy is one of the oldest surgical procedures and was first successfully performed in the late 19^th^ century. A tracheostomy is the formation of an artificial opening into the trachea between two adjacent rings of cartilage, with the aim of facilitate weaning from mechanical ventilation by decreasing anatomical dead space, prevention / treatment of retained tracheo-bronchial secretions or bypass upper airway obstruction.

Tracheostomy is a technique frequently performed on critically ill patients with respiratory failure who requiring prolonged mechanical ventilation. A retrospective nation-wide survey study reveal that the prevalence of tracheostomy was 10% in the ventilated patients under mechanical ventilation for more 24 hours. Most tracheostomies were performed during the 2nd week of ventilation. The frequency of tracheostomy varied widely according the hospitals (0–60 %) and was associated with the policy of hospitals to accept or refuse tracheostomized patients on their normal wards 11, and is predicted to become more common as demand for intensive care services increases 12,13.

Tracheostomy have several advantages respect translaryngeal endotracheal intubation: its might improve the weaning period through decreases respiration work and facilitate weaning by reducing airway resistance and need for less sedation 14–16. It has also been reported to lower the mortality rate 17–19 and to prevent complications of a prolonged orotracheal intubation 20. Other additional benefits are represented by facilitation of nursing care, more efficient suction of respiratory secretions and better patient’s comfort, swallowing and early phonation 21,22, although it is an invasive procedure with intrinsic technical risks 23–27.

The most frequent causes of death in tracheostomy patients are obstruction and haemorrhage. A variety of complications resulting from tracheostomy placement have been described. Intra operative complications: Bleeding (in < 5% of tracheostomies), damage to adjacent structures: (recurrent laryngeal nerves, major vessels), laceration of the esophagus, and false passage; 2) Post procedural early complications: Wound infection, subcutaneous emphysema, tube obstruction by mucus or blood clots; 3) Post procedural late complications: Cuff leak, tracheomalacia, tube occlusion, tube dislodgement, tracheo-esophageal fistula, tracheo-innominate fistula and the more common tracheal stenosis, due to narrowing of the trachea by stenotic granular tissue 21,24,28. The contraindications for tracheostomy are rare: severe hematologic and coagulation disorders, or soft tissues infections of the neck or anatomic abnormalities. It is relative contraindication the severe respiratory distress with severe hypoxemia 27.

The tracheostomy may be temporary or long term. A temporary tracheostomy tube will be inserted to maintain the patency of the airway and facilitate the weaning process due to the reduced airway resistance. This can be removed when the patient recovers. A temporary tracheostomy may become long term if the patient’s condition requires this. In non selected ICU-patients, tracheostomy allows earlier discharge of patients from the ICU, thus allowing better management of limited ICU resources 29,30, and may be associated with reduced mortality 17,31.

## RESPIRATORY INSUFICIENCY IN SPINAL CORD INJURY

Injury to cervical level of the spinal cord can lead to an impairment of the respiratory system by lung volumes decreases, changes in the breathing pattern and gas exchange alteration. As a result of the loss of diaphragmatic and/or intercostals muscle function, the higher and more complete the motor level of the injury, the respiratory system will become severely compromised following spinal cord injury (SCI). Interruption of the descending input from premotor neurons above the phrenic motoneurons located in the cervical spinal cord C3–C5 (phrenic nerve nucleus) results in immediate paralysis of the main inspiratory muscle, diaphragm. In C5 to C8 injury level there is paralysis of remaining inspiratory and expiratory muscles, which causes inefficient ventilation due to paradoxical respiration (chest wall distortion). This inefficiency contributes to the risk of respiratory muscle fatigue that causes reduction in vital capacity. There are also markedly impaired cough due to paralysis of expiratory muscles. Due to both factors are delayed insufficiency respiratory develops 5,32. Atelectasis, pneumonia and ventilatory failure are the most common complications during the acute phase of SCI, reason for also requires MV in many patients 3,6,7,33.

In the case of thoracic SCI injuries, respiratory failure will depend more on the associate injuries (severe thoracic trauma with pulmonary or myocardial contusion, severe pleural disease) or severe previous comorbidity (COPD, ischemic myocardiopathy, etc.), so considerations regarding tracheostomy implementation are those of the general critical patients 34–36. Also, in this patients its deteriorate bronchial secretions clearance, due to inefficacy of expiratory abdominal muscles, facilitates the respiratory insufficiency 37.

The indications for tracheostomy in the spinal cord injured patients, as in other patients with respiratory failure, include failure in extubation, upper airway obstruction, airway access for tracheobronchial secretions removal, avoidance of serious oropharyngeal and laryngeal injury from prolonged translaryngeal intubation, and, specially, expected prolonged dependence on mechanical ventilation 35. All these circumstances can be seen into tetraplegic patients.

The main aim of rehabilitation program in cervical SCI patients is enhance the residual activity of debilitate diaphragm, the accessory respiratory muscles recruitment as far as possible, stabilizing the rib cage (avoid paradoxical breathing) for improving both inspiratory and expiratory function, and strength training expiratory muscle function 38,39. This rehabilitation program usually takes long-time to achieve their goals, reason that the prolonged mechanical ventilation is standard measure in high level injured SCI patients.

In most cases this respiratory insufficiency is partially reversible duo to multiple factors, then the patient would weaning of mechanical ventilation after some time, more length the higher the level of the lesion. It is permanent if the level is above C4 sensory-motor and motor complete SCI (ASIA A + B) patients. In cases in which patients are unable to maintain adequate pulmonary ventilation, long-term mechanical ventilation support is indicated, and therefore the practice of tracheostomy 32,40.

An important clinical problem in SCI is weak cough, which causes retention of secretions during infections. As much as 50% of patients with a complete lesion of the cervical or thoracic spinal cord develop atelectasis or pneumonia within 30 days of the injury, with the peak incidence at 3–5 days 41. Methods for secretion clearance include chest physical therapy, and suctioning, cough assistance by forced compression of the abdomen, and mechanical insufflation-exsufflation 3,42, techniques that unfortunately not prevent the need of translaryngeal intubation in the main cases. This is the cause, besides the non-reversible decrease of vital capacity, for a rare utilization of non-invasive mechanical respiration without tracheostomy in these patients with complete cervical SCI 5,43.

After cervical SCI a great percentage of patients will require intubation and mechanical ventilation due to compromised respiratory function 44. Of these between 20.6 to 77% have required tracheostomy 9,45. In patients with traumatic complete cervical spinal cord injuries at C5 level and above required a definitive airways and tracheostomy, in patients whit complete injuries of C6–C8 50% required tracheostomy, however only 7% of incomplete injured patients required this procedure 40. A wide retrospective study by review a national databank of cervical SCI shown that 20.6% of cervical spinal cord injured patients were in need for tracheostomy implantation due to prolonged respiratory insufficiency 46.

In a retrospective study of the 175 patients with acute tetraplegia, 72 were tracheotomised. The need for a tracheotomy could be predicted in 73.31% based on the neurological level, the ASIA impairment scale and severity of accompanying injuries. According to this, the authors recommend that in patients with acute tetraplegia, primary tracheotomy is indicated immediately in all patients on C1–C3 level with ASIA scale A/B, at an early stage in the patients with C4–C6 injury level with ASIA scale A/B and accompanying injuries/accompanying illnesses and there were accompanying injuries/accompanying illnesses and/or there was a complex cervical spine trauma requiring a combined operative procedure and/or ventilation for more than 10 days was required 47. Others predictors for the need of tracheostomy in cervical spinal cord injured patients are the intubation in trauma scene or emergency department, higher severity of trauma, and maxillofacial fracture 46. In the others patients, an attempt at extubation should be done, given that respiratory-muscle training improves performance of the respiratory muscles and may decreases the mechanical ventilation duration, and implementing a tracheotomy did not affect the duration of treatment, duration of ventilation or length of stay in the intensive care unit (ICU). Following a failed attempt at extubation, early tracheotomy should be carried out 5,47.

In summary, SCI can causes impairment of respiratory muscles function, reduced vital capacity, ineffective cough and reduction in lung and chest wall compliance. Severely affected individuals may require assisted ventilation, in most patients prolonged, or definitive in some cases. Late partial recovery can occurs spontaneously. The eventual weaning depends on the extent of spontaneous partial recovery of respiratory-muscle performance, time since injury, antecedents of chest injury, and others respiratory co-morbidities. Due to the need of prolonged mechanical ventilation, ineffective cough which need its frequent removal, and delay on recovery, the tracheostomy technique as a high prevalence in SCI persons.

## TECHNIQUES: SURGICAL TRACHEOTOMY VERSUS PERCUTANEOUS DILATATIONAL TRACHEOTOMY

Tracheostomy is one of the traditional surgical procedures in ICU patients. In the last decades, percutaneous dilatational technique (PDT) has gained widespread acceptance as an alternative to surgical tracheostomy (ST). PDT is a less invasive bedside procedure. PDT was originally described by Ciaglia in 1985. In principle, the technique employs the use of serial dilators introduced into the trachea over a guide wire. Modifications to improve this technique have included design of special dilating forceps and use of a single-tapered dilator rather than multiplesized ones. Several commercial PDT kits are currently available, providing all of the necessary equipment for efficient and expedient application 27.

The development and rapid introduction of the PDT is due to it is technically easy to use, has a very low rate of complications it is minimally invasive, can be conducted with the neck in a neutral position 49–53, and can be safely performed at the bedside by experienced, skilled practitioners, with low complications rates which obviates the need to transport a critically ill patient to surgery room 54. The advantages are attributed to PDT are based on that the limited dissection results in less tissue damage, lowers the risk of bleeding and wound infection 55. A considerable advantage of PDT is the relative safety and convenience of performing the procedure at the bedside in the ICU 51. The contraindications to use of the percutaneous technique are: morbid obesity, unusual neck anatomy, repeated tracheostomy, need of high positive end-expiratory pressure, and severe coagulopathy 27.

Percutaneous tracheostomy was associated with a reduction in the incidence of clinically important wound infections but there was no clear difference in the incidence of clinically significant bleeding, major peri-procedural or long term complications, nevertheless few studies suggest a reduction in tracheal stenosis with PDT 54,56,57. The reasons for a reduced incidence of wound infection may be due to minimization of the local tissue damage with a dilatational technique, or may in part be due to a relative preservation of immune functions when minimally invasive techniques are used when compared to open techniques 55,58–60. Another possible advantages of PDT on SCI patients are a lower duration of ICU stay, and a lower incidence of pneumonia 57, whereas others authors have found no difference in the incidence of this complication 61,62. PDT allows a repeated tracheostomy is performed safely^63^.

Addition of bronchoscopy guidance to PDT may lead to a lower risk for peri-procedural complications, so bronchoscopy can be recommended as a simple and safe technique with the potential to avoid complications such as fausse route and posterior tracheal wall lacerations 55,56,64. In selected cases with difficulties in identifying anatomical landmarks, the ultrasound to PDT can be made 65. PDT should not be performed by an inexperienced practitioner when a free airway needs to be established urgently in case of difficult intubation.

Percutaneous dilatational tracheostomy, performed in the ICU, should be considered in this moment the procedure of choice for performing elective tracheostomies in critically ill adult patients both, general critical patients or SCI patients 27,54,57, when it is not contraindicated.

## TIMING

The prolonged intubation is the main risk factor for multiple complications. Local complications of translaryngeal intubation are more likely if tracheal intubation is continued for more than 2 weeks 24,66. The 86% of patients with subglotic stenosis had a history of prolonged tracheal intubation with a mean duration of ventilatory support of 17 days 67.

There has been a long debate on the optimal timing to place a tracheostomy in injured patients who require prolonged mechanical ventilation. The American College of Chest Physicians Consensus Conference on Artificial Airways in Patients Receiving Mechanical Ventilation issued the recommendation that tracheostomy is preferred if the need for mechanical ventilation is anticipated to be greater than 21 days 68, there is no evidence on timing and a practical decision is made by attending physician for every individual patient 69,70. New guidelines suggest that tracheostomy should be considered within the 7 first days when it becomes apparent that the patient will require prolonged ventilator support 27. A recent meta-analysis of the efficacy of early tracheostomy, reported that patients treated with early definitive airway placement could benefit from reduction of mechanical ventilation and ICU stay, while there were not changes in the rate of pneumonia or mortality 30,71,72.

Many authors have evidenced that early tracheostomy placement is associated to a marked reduction in ICU stay and/or in the duration of MV in patients admitted to an ICU with medical illnesses, polytrauma and in surgical and neurosurgical patients 19,29,30,73–77. In spite of that, there are also studies leading to controversial conclusions: neither in head injury trauma patients, nor in critical patients with thermal injuries, early tracheostomy has shown any clear advantage in shortening mechanical ventilation 78,79. In critically ill adult patients requiring prolonged mechanical ventilation, tracheotomy performed at an early stage (within the first week) may to lower the late laryngeal complications of prolonged translaryngeal intubation, shorten the duration of artificial ventilation and length of stay in intensive care 27.

Has been reported the incidence of pulmonary infections is reduced significantly following tracheotomy, and the rate of pulmonary infections increases with the duration of endotracheal intubation 4. Also it has been suggested that early tracheostomy might lower the rate of ventilatory associated pneumonia due to reduced colonization of the tracheobrochial tract. This point is still controversial as some authors reported a reduced rate of VAP with early tracheostomy 71,73,74,76,77,80,81,82, while other studies could not confirm these observations 75,78,79. Some authors evidenced how early tracheostomy also lowers ICU mortality rate 81, and the overall hospital mortality 83. Others reported a mortality rate reduction on a long term basis, but not on a short term 84. Although these observations were not confirmed by other authors that could not demonstrate any significant association between early tracheostomy and reduction of mortality rate in surgical patients, polytrauma patients or severe head injury patients 29, 85.

A meta-analysis has reported ET has no influence on mortality or pneumonia incidence in trauma patients 70, and another state the risk of pneumonia was unaltered by the timing of tracheostomy in unselected critically ill patients undergoing artificial ventilation 30.

Optimal timing to place tracheostomy in SCI, as well as in other critical illness, is still controversial for lack of evidence proving when it is more convenient to perform it 86. In a retrospective study in SCI we state an evident shortening in ICU stay and a marked reduction of the duration of MV for those patients who underwent early vs. late tracheostomy, in addition to lowering rates of severe orotracheal intubation complication as tracheal granulomas and tracheal stenosis 57,87.

In summary, early tracheotomy placement offers advantages in SCI patients they require prolonged mechanical ventilation, for shortening of mechanical ventilation, reducing ICU stay and probably decreases local late complications. Given of benefits suggested, we then recommend to implementing tracheostomy as soon as possible, preferable within the first week of ventilation.

## TRACHEOSTOMY AND CERVICAL SPINAL SURGERY

When a tracheostomy is performed after anterolateral cervical spine fixation surgery, the two incisions come into very close contact, which poses the potential risk of cross-contamination and therefore, of much-feared infection of the osteosynthetic wound. This is why, in daily clinical practice, there is a tendency to delay the procedure until the healing process following fixation surgery is completed or at a very prolonged translaryngeal intubation which could increase its complications 88. Besides the danger of wound infection, this explains why up until 1999, only 3 cases of tracheostomy were reported, to our knowledge, all three performed using the percutaneous dilation technique shortly after cervical vertebral fixation 89.

In recent years the development of PDT, minimally invasive, with low rate of complications and can be implemented in the bedside at ICU with the neck in a neutral position, has permitted this technique to be practiced shortly behind anterolateral cervical fixation surgery^53^.

Owing to the difficulty of hyperextending the neck, the presence of a recent scar, as a result of anterolateral cervical spine fixation, and the great difficulty associated with reintubation after failed extubation, performing a tracheostomy is complicated 90–92. It has a lower rate of complications due to the PDT minimizes damage to adjacent structures, as it is less aggressive to tissues than conventional surgical techniques. This is why some authors have already postulated that this should be the technique of choice when a tracheostomy has to be performed on a SCI patient who has previously undergone anterolateral vertebral fixation 53. The PDT is also starting to be employed shortly after osteosynthetic cervical surgery 89.

Retrospective studies shown very low infection rates in early tracheostomy after cervical spinal stabilization by anterolateral approach without surgical wound dehiscence, which supports that this procedure is safe and effective 45,93,94.

## DECANNULATION PROCESS AND STOMA CLOSURE

Tracheotomy removal in patients who were weaned from prolonged mechanical ventilation can be considered if they are clinically stable, do not have psychiatric disorders, have adequate swallowing and are able to expectorate. The criteria for tracheotomy decannulation are stable arterial blood gases, absence of distress, haemodynamic stability, absence of fever or active infection, PaCO2 < 60 mmHg, absence of delirium or psychiatric disorder, normal endoscopic examination or revealing stenotic lesion occupying <30% of the airway, adequate swallowing and able to expectorate 95.

Many patients recovering from long-term mechanical ventilatory support may have normal airways but limited ventilatory reserve due to neuromuscular disease or underlying chronic obstructive pulmonary disease. These patients may benefit from “downsizing” of the tracheostomy stoma using progressively smaller cuff-less tracheostomy tubes with intermittent capping using stomal obturators 27,96. Patients who are able to breathe around a capped cuffless tracheostomy tube most likely have adequate respiratory reserve and a sufficiently preserved native airway to tolerate decannulation. Some patients benefit from logopedic support.

Once successfully weaning, the tracheostomy remain patent until you consider that it will remain necessary aspirations of tracheobronchial secretions and the patient was capable of proper management. In this moment is possible to close the stoma, which is accomplished by removal of tracheal tube, the opening closed spontaneously. The wound spontaneously heals in less than 10 days in most cases. In our experience, the persistence of a tracheocutaneous fistula that needs surgical closure occurs in less than 5% of cases.
